# Mechanical asymmetries in gastrocnemius stiffness: shear-wave elastography insights into the biomechanics and injury susceptibility of calf muscle strain injuries

**DOI:** 10.1186/s13102-025-01340-x

**Published:** 2025-09-26

**Authors:** Yanhui Du, Zhe Pan, Yang Zhang, Fengxue Xuan, Haitao Yu, Bo Wang, Gaofeng Li, Guangchun Li, Weijing Zhang, Fei Chang

**Affiliations:** 1 Orthopaedic Medical Center, The Second Hospital of Jilin University, Changchun, Jilin Province China; 2 Departments of Orthopedics, Jilin Province People’s Hospital, Changchun, Jilin Province China; 3 Departments of Ultrasound, Jilin Province People’s Hospital, Changchun, Jilin Province China; 4 Departments of Ultrasound, The Second Hospital of Jilin University, Changchun, Jilin Province China

**Keywords:** Musculotendinous injury, Elastography, Dynamic load, Injury risk, Ultrasonography

## Abstract

**Background:**

“Tennis leg” injuries originate predominantly at the medial gastrocnemius (MG) musculotendinous junction (MTJ). Whether posture-dependent mechanical asymmetries between the MG and lateral gastrocnemius (LG) explain this susceptibility remains unclear. To quantify posture-specific stiffness of gastrocnemius muscle and MTJ with shear-wave elastography (SWE) and to identify mechanical signatures that may predispose the MG to strain.

**Methods:**

Ultrasound data were obtained from 22 calves, resulting in 44 samples, across three ankle positions: neutral, 15° plantarflexion, and 15° dorsiflexion, with the knee in an extended position. Young’s modulus was sampled at the muscle belly, the MTJ, and proximal 1 cm and distal 1 cm on both side; each value represented the mean of three trials. Independent t-tests were employed to assess side-to-side differences, while one-way ANOVA (Bonferroni-adjusted, α = 0.05) was utilized to evaluate posture effects.

**Results:**

Across neutral postures, the MG was stiffer than the LG at the muscle belly (*P* < 0.05), MTJ (*P* < 0.001), and at the distal 1 cm of MTJ. Dorsiflexion induced the highest stiffness values on both sides, with the MG demonstrating significantly greater stiffness than the LG (*P* < 0.05). Notably, despite its higher baseline stiffness, the MG showed a smaller contraction-induced percentage increase in stiffness compared to the LG at the MTJ in both plantarflexion (*P* < 0.05) and dorsiflexion (*P* < 0.05), indicating a high-stiffness/low-compliance (HNC) profile.

**Conclusion:**

Dorsiflexion markedly increases the stiffness of the MG while decreasing its proportion to enhance stiffness during contraction. This HNC behavior emphasizes stress at the in tennis-leg ruptures. SWE-derived HNC measurements may assist in identifying at-risk athletes and customizing preventive measures.

**Trial registration:**

Clinical trial number: not applicable.

## Introduction

Gastrocnemius strains, sometimes referred to as “tennis leg,” occur in approximately 3 out of every 1,000 match-hours and result in a return to play delay of three to six weeks; reinjury rates range from 19–31% [1–5]. High-resolution MRI and ultrasound now show that > 95% of these ruptures originate at the MG musculotendinous junction (MTJ), whereas isolated plantar-tendon tears are rare and up to 10% of suspected cases prove to be deep-vein thromboses [6]. The reason for the failure of this singular interface, while the lateral head remains predominantly unaffected, during standard knee-extension/ankle-dorsiflexion maneuvers is yet to be elucidated.

Shear-wave elastography (SWE) offers in-vivo, millimeter-scale mapping of muscle-tendon stiffness with excellent reliability (intraclass correlation coefficient > 0.85) [7–11]. Prior studies in SWE indicate that (1) baseline stiffness is greater in the MG compared to the lateral gastrocnemius (LG), and (2) passive ankle dorsiflexion significantly increases the shear modulus of the gastrocnemius [12–16]. Muscle stiffness, particularly in the gastrocnemius, has been proposed as a contributing factor to strain-related injuries [17]. Moreover, Weidlich found that stiffness imbalance is closely related to muscle and tendon injuries [18, 19]. The variations in stiffness surrounding the MTJ are essential to examine because of its function as a crucial structure connecting tendons and muscles. Nevertheless, this research focused primarily on resting or passive states, predominantly sampling the muscular bellies, and rarely compared both heads under functional stress. The asymmetry in stiffness related to posture at the medial versus the lateral MTJ, and its potential role in tennis-leg pathology, remains unexamined.

The present study therefore uses SWE to map Young’s modulus at the MTJ and musculotendinous regions of both gastrocnemius heads in three clinically relevant positions: neutral, 15° plantarflexion, and 15° dorsiflexion (knee extended).

We evaluated two predetermined hypotheses: H1: dorsiflexion disproportionately increases stiffness at the medial MTJ compared with the contralateral side; H2: this stiffness asymmetry mirrors the localization of tennis-leg injuries. By clarifying how posture amplifies mechanical imbalance within the gastrocnemius–Achilles complex, we aim to provide elastic evidence for the biomechanical properties of the medial MTJ.

## Methods

### Study design

This study was a cross-sectional experimental design, using SWE to quantitatively assess the stiffness changes in the medial and LG at different athletic postures, particularly focusing on the stiffness differences at the MTJ.

### Sample size

Sample size estimation was performed using G*Power software (V 3.1.9.2; Heinrich Heine University Düsseldorf, Germany) [20]. Preliminary data from 5 per group were used to estimate the effect size. For the independent samples t-test, with α = 0.05, power (1–β) = 0.95, and an estimated effect size = 1.57, an a priori power analysis indicated that a minimum total sample size of 24 would be required. Additionally, for the one-way ANOVA, assuming α = 0.05, power (1–β) = 0.95, and an effect size of 0.72, the required total sample size was determined to be 33.

### Participant recruitment

The study plans to recruit 11 healthy adult volunteers (22 calves; totaling 44 samples), all of whom have no history of lower limb muscle or tendon injuries. To exclude factors that may affect muscle stiffness measurements, participants must not have any neuromuscular diseases, joint disorders, or systemic diseases that could influence muscle function. All participants voluntarily agreed to participate in this study and signed informed consent forms before the experiment.

### Experimental equipment and measurement tools

Participants were instructed to refrain from strenuous exercise for one week prior to the experiment to minimize any effects on muscle stiffness. The stiffness of the medial and LG muscle bellies and the gastrocnemius MTJ was measured using an Aixplorer ultrasound scanner (Aixplorer, Aix-en-Provence, France) in both B-mode and SWE mode.

A line array probe model SL15-4 was used with a frequency range of 4–15 MHz. the B-mode was set to medium resolution (Res/Med), the mechanical index (MI) was kept at 1.0, the speed of sound was set to 1540 m/s, and the frame rate of the images was 12 Hz. the frequency of the shear wave was set to the standard mode (Std/2.0 Hz), and the elastography sample was set to medium intensity (M 1/2 Hz). The shear wave frequency was set to standard mode (Std/2.0 Hz), the elastography sampling was set to medium intensity (M 1/Med), and the shear wave scanning depth and sampling window were adjusted according to the measurement site, which was usually set in the range of 1.5–2.5 cm.

To ensure the reliability and accuracy of the measurements, all SWE assessments were performed by a physician with over ten years of experience in musculoskeletal ultrasound at a constant room temperature. During acquisition, image gain and contrast parameters are optimized for tissue depth and sharpness to ensure that tissue structure and elasticity changes in the imaged area are clearly discernible. All measurements were performed in triplicate on the same day, and the average value was used for analysis to minimize intra-operator variability and reduce measurement error.

### Region-of-Interest (ROI) placement

**Fig. 1:**
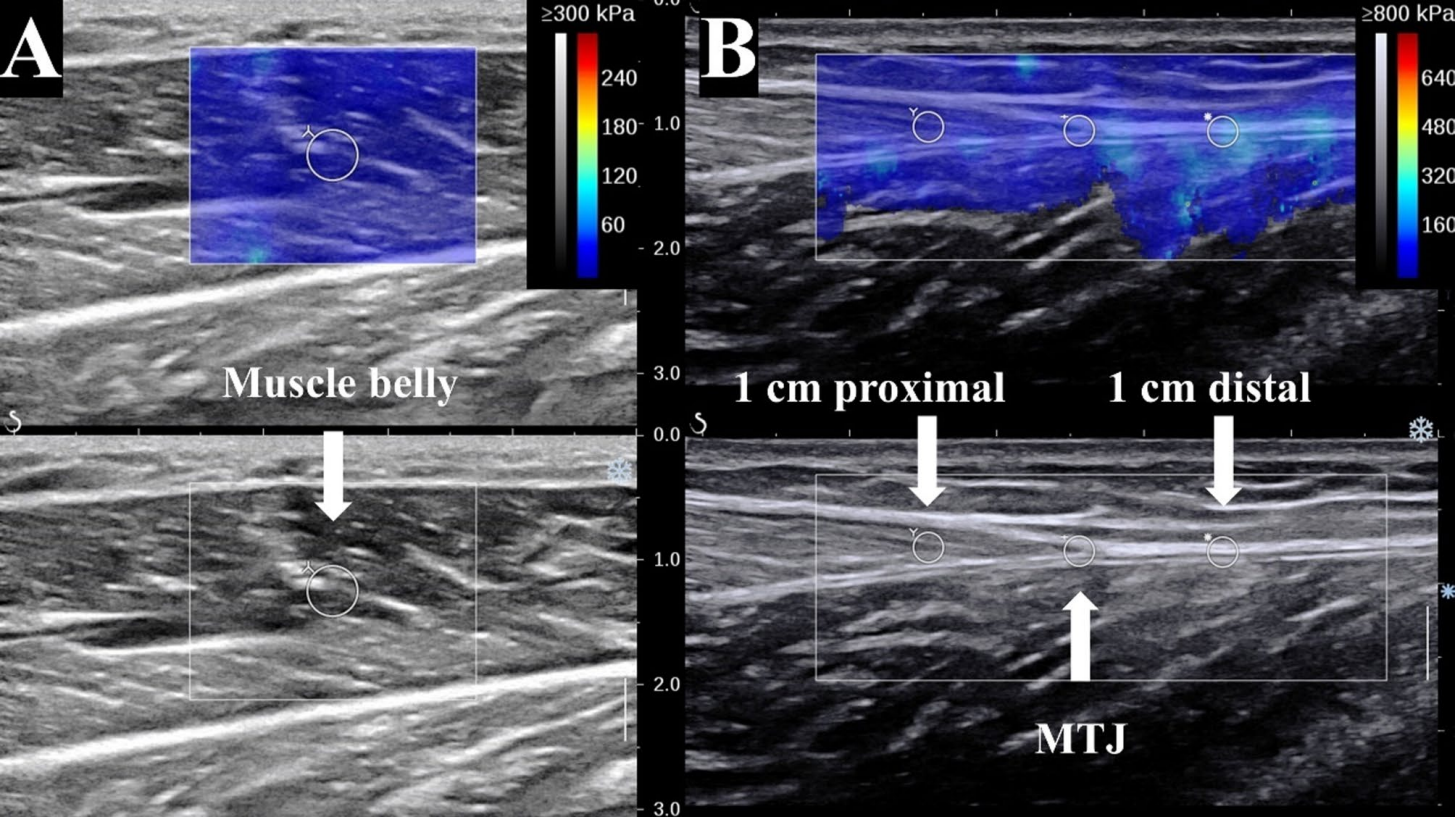
**A-B**: images of the ROI in the SWE. Muscle belly: Gastrocnemius muscle belly; MTJ: Musculotendinous junction; 1 cm proximal: 1 cm proximal to the MTJ;1 cm distal: 1 cm distal to the MTJ. The sampling area diameter of the gastrocnemius muscle belly is 4 mm (**A**), while the sampling area diameters of the musculotendinous junction, 1 cm proximal to the MTJ, and 1 cm distal to the MTJ are 2 mm (**B**)

For both the MG and LG heads, four ROIs were delineated in B-mode and automatically transferred to the elastogram: [16] Measurements were performed at four sites: 1 cm proximal to the MTJ, at the MTJ, and 1 cm distal to the MTJ (Fig. 1).

### Experimental procedures

Before the experiment, all participants performed light warm-up exercises to ensure their muscles were adequately prepared for activity and to minimize errors arising from excessive tension or insufficient preparation [13]. Three different postures were assessed, with stiffness measurements taken independently for each posture:

#### Neutral position

The participant lies prone with both knees extended, and the ankle in a natural, relaxed position.

#### Plantarflexion

The participant stands on a 15° inclined wedge board, holding the position for 10 s to ensure stability before the test begins.

#### Dorsiflexion

The participant stands on a 15° inclined wedge board, maintaining stability for 10 s before the test starts.

Stiffness measurements were performed under passive conditions without active muscle contractions. In the neutral position, participants lay in the prone position with the ankle naturally relaxed. For the dorsiflexion and plantarflexion, participants stood passively on an inclined platform set to the corresponding angles. Participants were instructed to remain relaxed and avoid any voluntary contractions during all measurements.

The dynamic contraction ratio is the stiffness during contracted state (plantarflexion or dorsiflexion) divided by the stiffness at rest.

### Statistical analysis

Intra-operator reliability analysis was conducted. Stiffness measurements for each participant were conducted three times by a single independent operator, and internal consistency was evaluated using the intraclass correlation coefficient (ICC). The ICC is typically interpreted as follows: values between 0 and 0.40 indicate poor reliability, 0.41 to 0.60 indicate moderate reliability, 0.61 to 0.79 indicate good reliability, and values from 0.80 to 1.00 indicate excellent reliability [21].

To ensure the assumptions of parametric testing were met, data normality was evaluated using the Shapiro-Wilk test prior to group comparisons. Descriptive statistics (M ± SD) were employed to analyze SWE data across various postures. Independent t-tests were conducted to compare stiffness between the medial and lateral sides within the same posture. One-way ANOVA was employed to analyze stiffness variations in the gastrocnemius and Achilles tendon across different postures, concentrating on stiffness alterations at various measurement locations (muscle belly, MTJ, and the 1 cm regions above and below the junction). Pairwise comparisons were adjusted utilizing the Bonferroni correction for *p*-values. The effect sizes (ES), measured by Cohen’s d, were computed to assess the differences between evaluations. An ES of less than 0.20 was considered small, values between 0.20 and 0.50 were classified as medium, and values greater than 0.80 were regarded as large [22]. Statistical analyses utilized SPSS 27, establishing a significance threshold at *α* < 0.05.

## Results

The baseline characteristics is described in Table 1. The overall measurement reliability was found to be excellent, with ICCs of 0.80 or higher for all conditions, except for the proximal 1 cm of the MTJ in the neutral position (ICC = 0.753) and dorsiflexion (ICC = 0.798; Table 2) was good reliability.

**Table 1:** Basic and physical activity characteristics of participator

Variable	Mean ± SD
N	11
Age, years	32.45 ± 5.12
Height, cm	172.91 ± 4.12
Weight, kg	71.64 ± 4.79
Gender, F/M	6 / 5
Activity Level, h	4.6 ± 1.65
Leg Dominance, L/R	5 / 6

**Table 2:** Intra-rater reliability of SWE measurements across three consecutive trials (ICC), standard error of measurement (SEM), and 95% confidence level (MDC95)

	Posture	Part	ICC	95%Cl	SEM	MDC95	
Medial	Neutral	Gastroc Belly	0.979	(0.88,0.972)	0.09	0.25	
		Proximal 1 cm to MTJ	0.753	(0.498,0.89)	0.28	0.77	
		MTJ	0.909	(0.815,0.959)	0.20	0.55	
		Distal 1 cm to MTJ	0.953	(0.905,0.979)	0.10	0.27	
	Plantarflexion	Gastroc Belly	0.935	(0.869,0.971)	0.16	0.45	
		Proximal 1 cm to MTJ	0.884	(0.763,0.948)	0.16	0.45	
		MTJ	0.804	(0.601,0.912)	0.25	0.69	
		Distal 1 cm to MTJ	0.856	(0.708,0.936)	0.22	0.62	
	Dorsiflexion	Gastroc Belly	0.973	(0.946,0.988)	0.11	0.30	
		Proximal 1 cm to MTJ	0.798	(0.59,0.91)	0.27	0.76	
		MTJ	0.926	(0.851,0.967)	0.14	0.39	
		Distal 1 cm to MTJ	0.94	(0.878,0.973)	0.18	0.50	
Lateral	Neutral	Gastroc Belly	0.983	(0.966,0.9993)	0.08	0.22	
		Proximal 1 cm to MTJ	0.972	(0.944,0.988)	0.09	0.26	
		MTJ	0.976	(0.951,0.989)	0.05	0.14	
		Distal 1 cm to MTJ	0.972	(0.943,0.987)	0.12	0.32	
	Plantarflexion	Gastroc Belly	0.966	(0.93,0.985)	0.12	0.34	
		Proximal 1 cm to MTJ	0.938	(0.875,0.972)	0.14	0.39	
		MTJ	0.987	(0.974,0.994)	0.07	0.19	
		Distal 1 cm to MTJ	0.985	(0.97,0.993)	0.08	0.22	
	Dorsiflexion	Gastroc Belly	0.969	(0.938,0.986)	0.11	0.30	
		Proximal 1 cm to MTJ	0.959	(0.917,0.982)	0.15	0.41	
		MTJ	0.984	(0.968,0.993)	0.09	0.24	
		Distal 1 cm to MTJ	0.96	(0.918,0.982)	0.12	0.34	

**Fig. 2:**
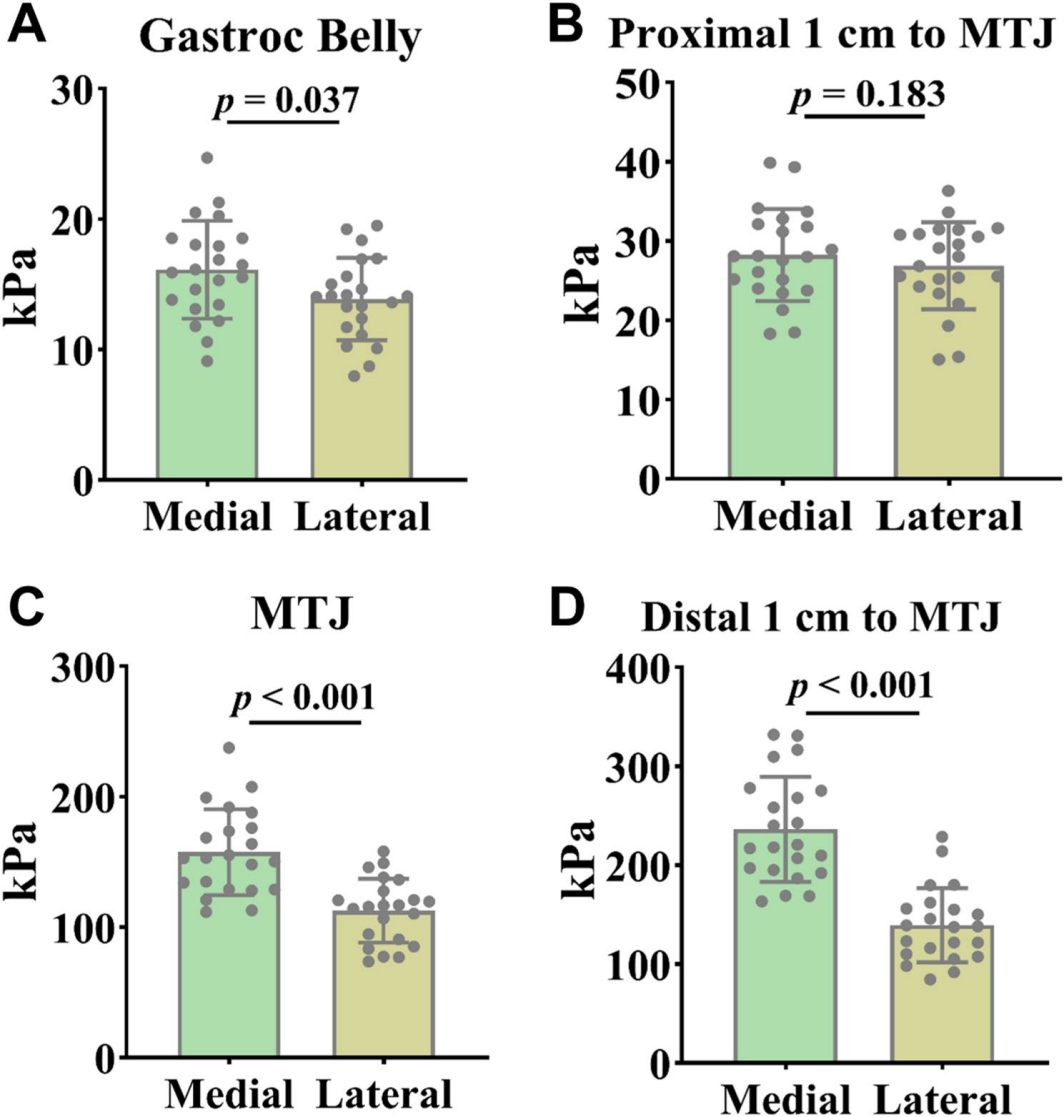
**A-D**: Results of the resting neutral position test. Gastroc belly: Gastrocnemius muscle belly; MTJ: Musculotendinous junction

In the neutral position, the medial side of the gastrocnemius muscle belly demonstrated significantly greater stiffness compared to the lateral side (Fig. 2a, *P* = 0.037, ES = 0.65, CI 95% = 0.04–1.25). No significant difference in stiffness was noted 1 cm proximal to the MTJ (Fig. 2b, *P* > 0.05, ES = 0.41, CI 95% = -0.2-1). At the MTJ, the medial side exhibited significantly greater stiffness than the lateral side (Fig. 2c, *P* < 0.001, ES = 1.55, CI 95% = 0.87–2.22). At a distance of 1 cm distal to the MTJ, the medial side demonstrated significantly greater stiffness compared to the lateral side (Fig. 2d, *P* < 0.001, ES = 2.11, CI 95% = 1.36–2.84).

**Fig. 3:**
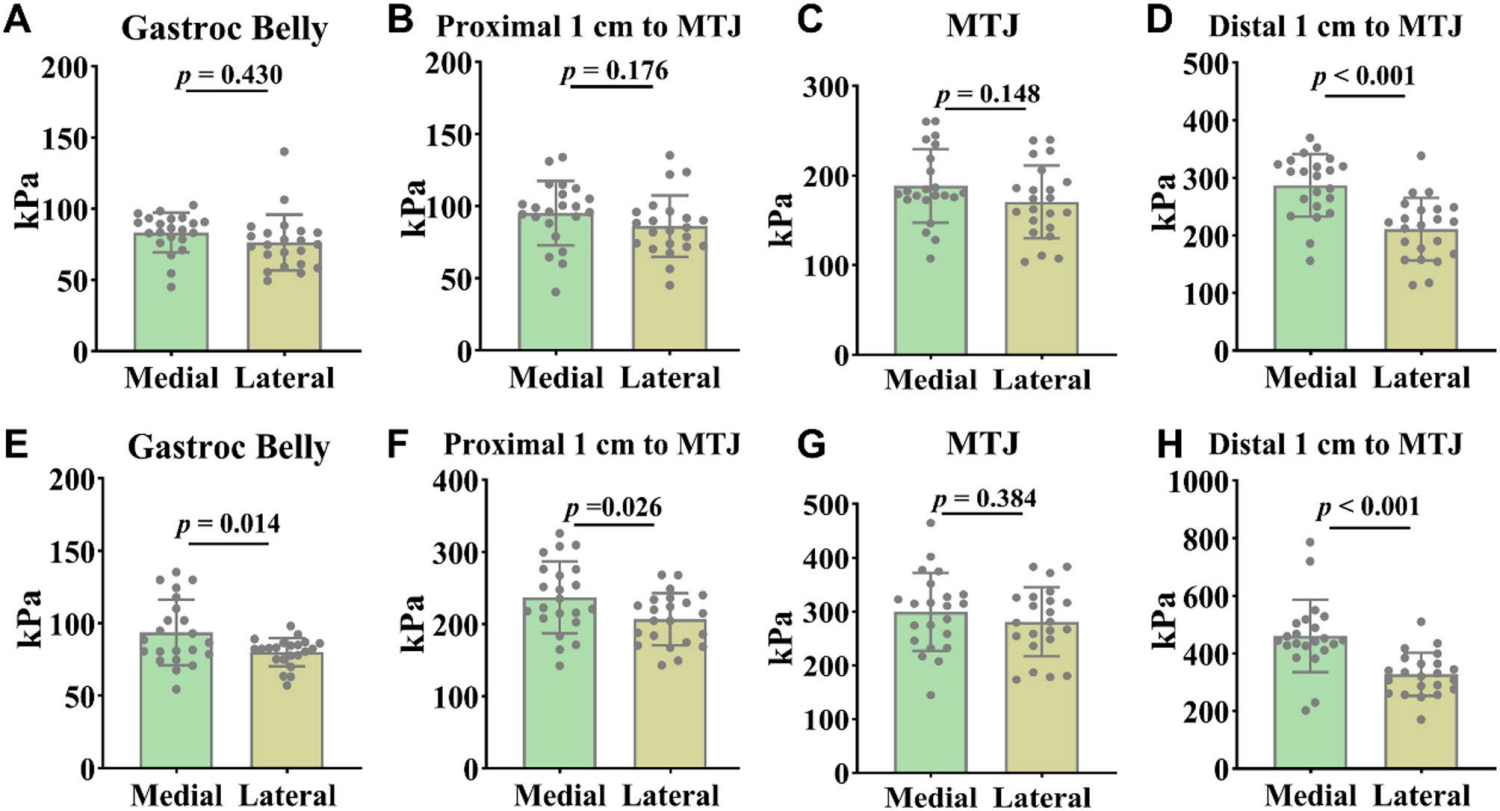
**A-D**: Results of the ankle plantarflexion test at 15°. E-H: Results of the ankle dorsiflexion test at 15°. Gastroc belly: Gastrocnemius muscle belly; MTJ: Musculotendinous junction

During both ankle plantarflexion and dorsiflexion, the medial side demonstrated significantly greater stiffness than the lateral side at certain locations Specifically, under plantarflexion, this difference was observed 1 cm distal to the MTJ (Fig. 3d, *P* < 0.001, ES = 1.40, CI 95% = 0.73–2.05), with no significant differences at the other sites (Fig. 3a, *P* > 0.05, ES = 0.24, CI 95% = -0.35-0.83 ; **3b**, *P* > 0.05, ES = 0.42, CI 95% = -0.19-1.01; **3c**, *P* > 0.05, ES = 0.44, CI 95% = -0.16-1.02;). Under dorsiflexion, significantly greater medial stiffness was found at the gastrocnemius muscle belly, 1 cm proximal to the MTJ, and 1 cm distal to the MTJ (Fig. 3e, *P* < 0.05, ES = 0.78, CI 95% = 0.16–1.38 ; **f**, *P* < 0.05, ES = -0.70, CI 95% = -1.30-0.82 ; and **h**, *P* < 0.05, ES = 1.29, CI 95% = 0.63–1.94 ;), while no significant difference was observed at the MTJ itself (Fig. 3g, *P* > 0.05, ES = 0.27, CI 95% = -0.33-0.86).

**Fig. 4:**
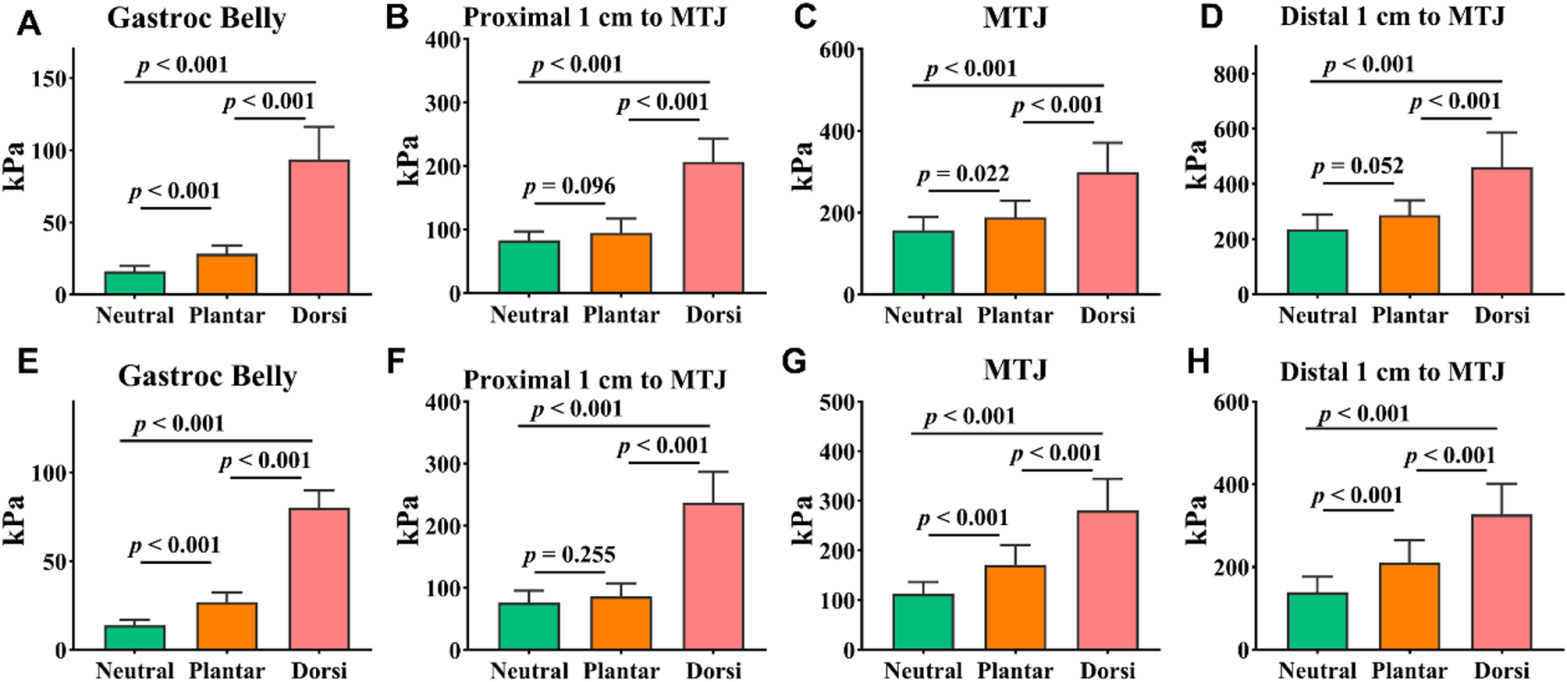
**A-D**: Results of the four medial test positions. **E-H**: Results of the four lateral test positions. Neutral: Neutral position; Plantar: Ankle plantarflexion at 15°; Dorsi: Ankle dorsiflexion at 15°. Gastroc belly: Gastrocnemius muscle belly; MTJ: Musculotendinous junction

Analysis of SWE across various positions indicated that dorsiflexion produced the highest stiffness values for both the medial and lateral sides at all test positions, in comparison to neutral and plantarflexion (Fig. 4, *P* < 0.001). For the medial side, the plantarflexion stiffness was significantly greater than at the neutral position at both the gastrocnemius muscle belly and the MTJ (Fig. 4a, *P* < 0.05, ES = -2.54, CI 95% = -1.74-3.33; and c, *P* < 0.05, ES = -0.86, CI 95% = -0.24-1.48 ;). On the lateral side, stiffness during plantarflexion were significantly higher than those in the neutral position at the gastrocnemius muscle belly, MTJ, and 1 cm distal to the MTJ (Fig. 4e, *P* < 0.01, ES = -2.97, CI 95% = -2.11-3.82; g, *P* < 0.01, ES = -1.77, CI 95% = -1.07-2.47; and h, *P* < 0.01, ES = -1.57, CI 95% = -0.89-2.24).

**Fig. 5:**
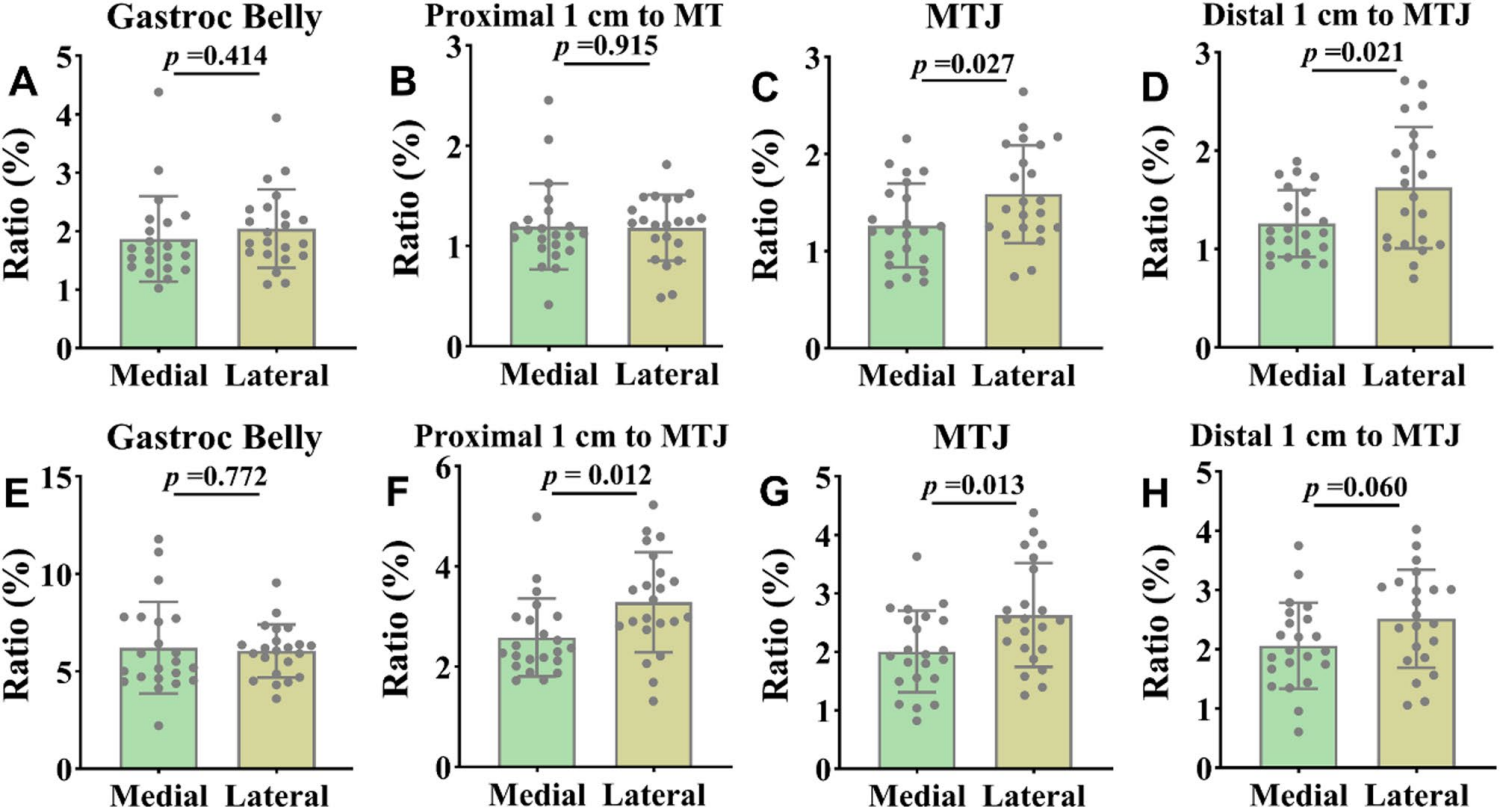
**A-D**: Results of the elastic potential energy test at ankle plantarflexion at 15° (plantarflexion divided by the resting SWE value). **E-H**: Results of the elastic potential energy test at ankle dorsiflexion at 15° (dorsiflexion divided by the resting SWE value). Ratio: The ratio of SWE values during contraction to the SWE value at rest. Gastroc belly: Gastrocnemius muscle belly; MTJ: Musculotendinous junction

The contraction ratio were no significant changes between the MG and LG during plantarflexion and dorsiflexion at the muscle belly (Fig. 5a, *P* > 0.05, ES = -0.25, CI 95% = -0.84-0.35; **and e**, *P* > 0.05, ES = 0.09, CI 95% = -0.50-0.68). At 1 cm proximal to the MTJ, no significant difference was noted during plantarflexion (Fig. 5b, *P* > 0.05, ES = 0.03, CI 95% = -0.56-0.62); however, during dorsiflexion, the medial side showed a significantly lower ratio compared to the lateral side (Fig. 5f, *P* = 0.012, ES = -0.79, CI 95% = -1.40-0.17). The medial side at the MTJ exhibited a significantly lower contraction ratio compared to the lateral side during plantarflexion (Fig. 5c, *P* = 0.027, ES = -0.69, CI 95% = -1.30-0.08), and this disparity was similarly noted during dorsiflexion (Fig. 5g, *P* = 0.013, ES = -0.78, CI 95% = -1.39-0.17). At 1 cm distal to the MTJ, the ratio on the medial side was significantly lower than that on the lateral side during plantarflexion (Fig. 5d, *P* = 0.021, ES = -0.72, CI 95% = -1.34-0.12), however, no significant difference was observed during dorsiflexion (Fig. 5h, *P* > 0.05, ES = -0.58, CI 95% = -1.18-0.02).

## Discussion

Our study mapped shear-modulus distributions in eight gastrocnemius regions with the ankle in neutral, plantar-flexed, and dorsi-flexed positions. Two hypotheses were tested: (1) the MG is intrinsically stiffer than the LG at rest and during passive stretch; and (2) this stiffness asymmetry reflects the typical localization of tennis-leg injuries. Both were confirmed: static stiffness was consistently greater in the MG across the muscle belly, the MTJ, and adjacent regions (1 cm proximal and distal to the MTJ). This asymmetry was most evident at the medial MTJ during dorsiflexion, aligning with the most commonly reported location of musculotendinous rupture in tennis leg. The findings indicate that dorsiflexed posture exacerbates the mechanical imbalance between the MG and LG, especially at the medial MTJ, which may increase the risk of injury in this area.

### Differences in stiffness and adaptability between the MG and LG

Across neutral, plantarflexed, and dorsiflexed postures, the elasticity of the MG was consistently greater than that of the LG. At rest, this difference was most pronounced at the MTJ and its distal segment. Morphologically, the MG possesses a larger muscle-belly volume and physiological cross-sectional area, and it contributes more than 70% of plantar-flexion force generation [23]. Its predominance of fast-twitch fibres and thicker aponeurosis render the MG inherently stiffer than the LG [23–25]. Functionally, therefore, the MG supplies explosive power, whereas the LG provides postural support and mediolateral stability, a division of labour also reported by Vieira et al. [26, 27].

The findings indicate that the MG muscle exhibits greater resting stiffness and a lower contraction-stiffness ratio, resulting in reduced flexibility during dynamic movements despite its stiffer state at rest. This HNC profile can be comprehended through three interrelated perspectives. Initially, a denominator effect occurs, indicating that a high resting modulus increases the denominator of the ratio. This results in the absolute SWE remaining constant, while the relative change is diminished [28]. The aponeurosis of the MG is larger and thicker, leading to a reduced architectural gear ratio. This reduces the capacity of fibres to rotate and move longitudinally, thereby inhibiting dynamic stiffness modulation [29]. Third, surface-EMG studies indicate that rapid plantar flexion engages a greater number of LG motor units, whereas MG motor units achieve their peak activation earlier, resulting in limited capacity for subsequent compliance adjustment [25]. This information is crucial for understanding the mechanisms behind tennis leg injuries, which frequently result from intense muscle stretching and contraction, particularly during high-intensity activities [1, 6, 16]. The MG is more susceptible to injury due to its inability to rapidly engage and alter compliance.

Previous work has shown that joint-angle manipulations dynamically redistribute tension within the gastrocnemius–Achilles complex [9, 30]; our decision to cap plantar-flexion at 15° minimized confounding from inversion [31]. Although elevated stiffness benefits force transmission, it can also predispose tissue to strain injury when load changes rapidly. Yoshida et al. reported that a higher MTJ shear modulus predicts a greater risk of gastrocnemius strain [16, 32]. The combination of their findings with our HNC observation suggests that the MG, because of its limited capacity to dissipate sudden eccentric energy, is susceptible to uneven stress concentration within the MTJ complex. This provides a crucial biomechanical rationale for its heightened vulnerability to tears, strains, and tendinopathy during high-load or sudden directional movements.

### Dorsiflexion position, dynamic load adaptation, and tennis leg injury mechanisms

With the knee extended and the ankle dorsiflexed, the gastrocnemius–Achilles unit reaches its longest functional length [33]. In this posture we observed a marked rise in shear modulus on the medial side at the muscle belly and at sites 1 cm proximal and distal to the MTJ, yet no further increase at the MTJ itself—an elastic pattern that agrees with earlier ultrasound work [8, 34]. Initially, this appears paradoxical; however, it aligns with the HNC model: the MTJ is inherently “hard,” thus further elongation primarily increases tension in the more compliant segments directly adjacent to it.

Dorsiflexion biomechanically shifts the center of pressure anteriorly, increasing the eccentric demand on the MG. Due to the shorter length and reduced elongation capacity of MG fascicles, the same stride or split-step results in greater fiber strain compared to the longer-fascicled LG [23]. The combination of (1) elevated baseline tension, (2) inadequate dynamic compliance, and (3) substantial eccentric loading concentrates stress within the medial MTJ complex. Finite-element and in-vivo strain-mapping studies identify this very region as the peak longitudinal-shear interface during forced dorsiflexion, which explains why acute “tennis-leg” ruptures occur overwhelmingly on the medial side of the MTJ [6]. Our data therefore provide direct, quantitative evidence that the MG’s limited adaptive reserve in dorsiflexion is a mechanistic link between posture, load transfer, and the characteristic injury pattern.

### Clinical recommendations: SWE-based monitoring and training regulation

Our findings revealed a consistent stiffness asymmetry between the medial and lateral gastrocnemius, particularly at the medial MTJ during dorsiflexion, a region prone to tennis leg injuries. These posture-dependent stiffness changes suggest that specific joint positions may amplify mechanical imbalances within the gastrocnemius–Achilles complex. Clinically, this highlights the potential of SWE as a non-invasive tool to identify individuals at elevated risk of strain injuries based on localized stiffness patterns.

### Limitations

Several constraints temper the generalizability of our findings. **First**, the cohort was small (*n* = 44) and comprised healthy adults only. Although an *a* priori power calculation and excellent intra-class correlation coefficients support the statistical adequacy of the data, larger and more heterogeneous samples—especially athletes with a history of MTJ injury—are needed to refine cut-off values for the HNC index. **Second**, the study employed a cross-sectional design; therefore, it was not possible to investigate the temporal adaptation of stiffness or the correlation between fluctuations in the HNC profile and injury incidence. Longitudinal monitoring throughout the season would elucidate these temporal relationships **Third**, SWE captures shear modulus along a single imaging plane and assumes tissue isotropy; factors such as probe orientation, fascicle pinnation, and transient fluid shifts may have influenced absolute values despite our stringent positioning protocol. Additionally, the gel’s properties result in the gel layer on the skin surface not being consistently apparent. This raises the likelihood that probe-induced compression may not have been entirely mitigated. While our study shows a reliable ICC, additional control of this potential error could improve the reliability of repeated measurements. **Finally**, our study focused solely on static postures; subsequent research should investigate dynamic tasks (e.g., landing, cutting) to determine whether the hard-but-non-compliant behavior persists in sport-specific contexts. Improving SWE-guided screening and intervention clinical translation requires addressing these challenges.

## Conclusion

This study confirms a significant stiffness asymmetry within the gastrocnemius–Achilles complex: the MG is consistently stiffer than the LG across neutral, plantarflexed, and dorsiflexed positions, with the disparity most pronounced at the MTJ during dorsiflexion. This localized stiffness pattern aligns with the typical site of tennis-leg injuries, supporting the hypothesis that posture-dependent mechanical imbalance contributes to medial MTJ vulnerability. However, it is important to note that while the association between stiffness asymmetry and injury site is plausible, further longitudinal studies are needed to establish a causal relationship.

## Data Availability

Data are available under reasonable request to the corresponding author.

## Abbreviations

HNC: High-stiffness/low-compliance
LG: Lateral gastrocnemius
MG: Medial gastrocnemius
MTJ: Musculotendinous junction
SWE: Shear-wave elastography
